# Behavioral health providers' perspectives of delivering behavioral health services in primary care: a qualitative analysis

**DOI:** 10.1186/1472-6963-12-337

**Published:** 2012-09-25

**Authors:** Gregory P Beehler, Laura O Wray

**Affiliations:** 1 VA Center for Integrated Healthcare, VA WNY Healthcare System, Buffalo, NY, USA; 2 School of Nursing, University at Buffalo, The State University of New York, Buffalo, NY, USA; 3 School of Public Health and Health Professions, The State University of New York, Buffalo, NY, USA; 4 Graduate School of Education, The State University of New York, Buffalo, NY, USA; 5 School of Medicine, University at Buffalo, The State University of New York, Buffalo, NY, USA

**Keywords:** Primary health care, Mental health services, Veterans, Program evaluation, Qualitative research

## Abstract

**Background:**

Co-located, collaborative care (CCC) is one component of VA’s model of Integrated Primary Care that embeds behavioral health providers (BHPs) into primary care clinics to treat commonly occurring mental health concerns among Veterans. Key features of the CCC model include time-limited, brief treatments (up to 6 encounters of 30 minutes each) and emphasis on multi-dimensional functional assessment. Although CCC is a mandated model of care, the barriers and facilitators to implementing this approach as identified from the perspective of BHPs have not been previously identified.

**Methods:**

This secondary data analysis used interview data captured as part of a quality improvement project in 2008. Fourteen BHPs (48% of providers in a regional VA network) agreed to participate in a 30-minute, semi-structured phone interview. The interview included questions about their perceived role as a CCC provider, depiction of usual practice styles and behaviors, and perceptions of typical barriers and facilitators to providing behavioral healthcare to Veterans in CCC. Interviews were transcribed verbatim into a text database and analyzed using grounded theory.

**Results:**

Six main categories emerged from the analysis: (a) Working in the VA Context, (b) Managing Access to Care on the Front Line, (c) Assessing a Care Trajectory, (d) Developing a Local Integrated Model, (e) Working in Collaborative Teams, and (f) Being a Behavioral Health Generalist. These categories pointed to system, clinic, and provider level factors that impacted BHP’s role and ability to implement CCC. Across categories, participants identified ways in which they provided Veteran-centered care within variable environments.

**Conclusions:**

This study provided a contextualized account of the experiences of BHP’s in CCC. Results suggest that these providers play a multifaceted role in delivering clinical services to Veterans while also acting as an interdependent component of the larger VA behavioral health and primary care systems. Based on the inherent challenges of enacting this role, BHPs in CCC may benefit from additional implementation support in their effort to promote health care integration and to increase access to patient-centered care in their local clinics.

## Background

The Department of Veterans Affairs (VA) has made significant efforts to integrate mental health providers and services into primary care [1,2]. The goals of this Primary Care-Mental Health Integration (PC-MHI) have been to improve access to mental health services, improve detection of mental health problems, improve quality of care by increasing collaboration between mental health and primary care providers (PCPs), decrease the number of patients lost to care during the referral process, destigmatize mental health care, and provide Veterans with service in a format that they find most desirable [2]. PC-MHI has a particular focus on improving the screening and treatment of depression, anxiety disorders, alcohol misuse, and posttraumatic stress disorder in primary care [3]. Although considerable progress has been made [3,4], whether or not these goals can be fully attained will be determined by the VA’s success in implementing the integration. As the VA is the nation’s largest health care system, a transformation of this nature presents considerable challenges, but also a wealth of opportunity to discover what strategies and techniques result in the most optimal implementation outcomes.

VA has operationalized the integration of mental health into primary care by designating two key components to establish in all larger primary care clinics [2]. These two components are care management and co-located, collaborative care (CCC) [5]. Although definitions of these forms of care vary considerably, in general, care management includes algorithm-based, protocol driven assessment and monitoring of symptoms, patient education, and motivational enhancement for specific mental health disorders, such as depression [6,7]. In contrast, CCC embeds an independent behavioral health provider (BHP) into the primary care clinic where he or she is expected to work collaboratively to support the PCP's care of the Veteran around a broad range of mental health diagnoses and behavioral concerns [8-10]. BHPs, therefore, are available to offer immediate follow-up of VA mandated mental health screens (e.g., depression, alcohol, posttraumatic stress disorder) with brief assessments and interventions for a wide variety of mental health and health behavior concerns. Many VA facilities initiated PC-MHI programming by implementing a single component, either CCC or care management [3]. The majority of VA Medical Centers (VAMCs) and large Community Based Outpatient Clinics (CBOCs) responding to a national administrative survey in 2010 reported providing either CCC alone or CCC blended with care management services [4].

**Figure 1:**
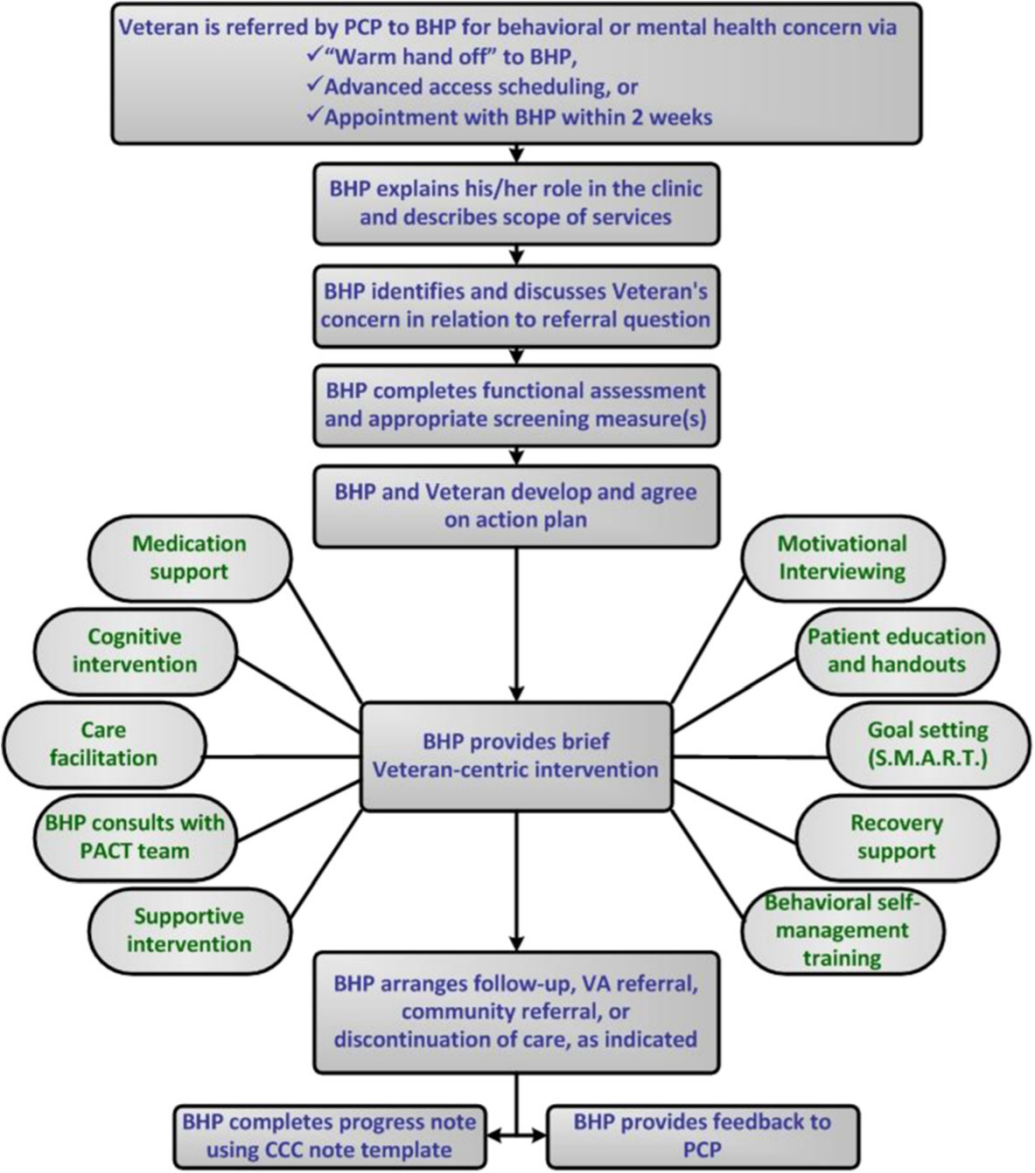
Processes of a Co-located, Collaborative Care encounter in VISN 2.

Implementation of depression care management services in VA primary care settings has received significant attention in the scientific literature [6,7,11-13] but comparatively few publications have focused on the implementation of CCC in VA. One example of CCC in VA is the regionally-based program in the Veteran's Integrated Service Network 2, VA Health Care Upstate New York (VISN 2). In this model, BHPs are embedded in every primary care clinic to help PCPs recognize, assess, and manage mental health conditions. Several key program features include (a) brief behavioral health sessions of about 30 minutes per encounter with 3–4 encounters per episode of care, (b) open clinic scheduling allowing same-day access, and (c) "curbside" consultation in which BHPs and PCPs could easily and informally share patient information to direct a course of care. The specific processes of a CCC encounter are represented graphically in Figure 1. In evaluation of the VISN 2 CCC model, Funderburk and colleagues [14] found that, as prescribed by the program, BHPs addressed a wide variety of disorders and conditions and provided support for referrals and liaison to specialty care services when appropriate. Overall, PCPs, BHPs, and patients surveyed expressed satisfaction with the program. Additionally, the authors found that the model had been altered from the planned implementation but they were unable to determine whether these variances reflected practical and necessary changes to the program or limitations caused by unidentified obstacles.

To date, the above authors and others have focused primarily on using quantitative methods to describe programs and how these programs improve recognition of mental health diagnoses [15], access to mental health care [9,16] and satisfaction with that care [14]. Although qualitative methods have been applied less frequently to the evaluation of PC-MHI, qualitative inquiry is nonetheless highly valuable for understanding the health care context and experiences of health care providers [17]. No authors to date have attempted to understand the barriers and facilitators to implementing and delivering CCC services by using qualitative methods to describe the experience of the staff providing CCC. Without firsthand accounts from BHPs, it is difficult to accurately characterize and understand their practices and clinic environments. To this end, a qualitative analysis was conducted using semi-structured interview data previously collected from CCC BHPs as part of a program evaluation project in VISN 2 completed in 2008. Conducting a qualitative analysis using these previously collected qualitative data provided the opportunity to more fully utilize this information to (a) understand how BHPs in primary care conceptualized their role in clinics providing CCC, and (b) uncover BHP-identified barriers and facilitators to providing Veteran-centered care.

## Methods and procedures

This report describes a secondary analysis of data that were collected as part of an integrated care program evaluation (final report available from second author) undertaken in conjunction with a quality improvement process in VISN 2 in 2008 [18]. Because care management services in the VISN were in the earliest stages of implementation, the program evaluation focused only on CCC services. Prior to beginning the qualitative analysis described below, we sought and received approval from the VA Western New York Healthcare System Institutional Review Board to use these de-identified data for research purposes.

### VISN 2 program evaluation participants

At the time of the program evaluation, 29 BHPs were employed across 34 sites in VA VISN 2 which encompasses upstate New York and parts of northern Pennsylvania. These sites included 5 VAMCs and 29 CBOCs. CBOCs are outpatient primary care centers located in satellite offices of parent VAMCs. In an effort to engage front line providers as efficiently as possible, BHPs were emailed an introduction to the project aims with an invitation to participate. Three follow-up emails were used over the course of two months to encourage participation, each noting progress in recruitment to date. Because of the relatively small number of BHPs in the VISN, the goal to contact and encourage participation from as many providers as possible was used to ensure a sufficiently large sample size. In total, 14 BHPs (48%) provided verbal consent to participate in a digitally recorded interview. Participants were primarily social workers (n = 8), as well as psychologists (n = 5) and a psychiatric nurse practitioner (n = 1). The majority of BHPs worked at CBOCs (n = 10); two worked at VAMCs and one BHP worked in both settings. Because some BHPs worked at multiple sites, a total of 15 primary care clinics were represented. BHPs length of employment at the VA ranged from 4 months to 27 years.

### Interview schedule, procedures, and data management

The interview schedule was developed to represent three main topics: BHP perceptions of their role in the primary care clinic, BHP depiction of usual clinical practice and behaviors, and BHP perceptions of barriers and facilitators to working in the CCC model, including site and patient characteristics. Interviews began with the grand tour question, “Tell me about your role as a provider in the clinic.” Probes and prompts were included to promote deeper exploration of the main items. BHPs who replied to the invitation to participate were contacted by email to schedule a telephone interview between July and August of 2008. Trained interviewers conducted the interview which lasted for about 30 minutes. These relatively brief interview times were adopted to ensure that BHPs were able to coordinate their participation in the program evaluation while managing a full day of clinical duties. Digitally recorded interviews were transcribed verbatim into a text database with all provider, clinic, and patient identifiers removed.

### Analytic approach

A grounded theory approach was used to analyze the BHP narratives [19-21]. Although there are numerous approaches to conducting qualitative analysis, we chose grounded theory because of its atheoretical, discovery-oriented approach that appeared well-suited to examining previously undocumented experiences of BHPs in CCC settings. The interpretive nature of a grounded theory provides analytic results that are both "grounded" in the data, but conceptualized at an abstract level. For the current study, transcripts were analyzed line-by-line to ensure that the data were exhausted by inductively coding the smallest relevant units of text, resulting in over 1,100 initial (or open) codes. Focusing on small units of text ensured that we maximized the utility of the dataset for this secondary analysis. Codes were compared within and across interviews, with conceptually similar codes clustered together to rebuild the data into preliminary categories. As analysis proceeded, 16 preliminary categories were refined or subsumed, resulting in six final categories, or main themes. Illustrative quotes were used to enhance the descriptive vividness of categories. Analytic memos were written throughout, providing both a record of the data analysis process and a framework for explaining the results of the analysis. ATLAS.ti software was used to facilitate the data analysis process [22].

## Results

The six categories that characterized the BHPs conceptualizations of barriers and facilitators to implementing CCC are summarized in Table 1 and point to the multi-level nature CCC implementation. *Working in the VA Context* and *Managing Access to Care on the Front Line* described health care system factors that impacted the BHPs role and ability to implement CCC. *Assessing a Care Trajectory* and *Developing a Local Integrated Model* depicted local systemic and clinic level factors that impacted CCC implementation. *Working in Collaborative Teams* and *Being a Behavioral Health Generalist* depicted relevant clinic and provider factors in relation to CCC implementation.

**Table 1:** Summary of categories derived from qualitative analysis of Behavioral Health Provider narratives

**System, clinic, and provider level factors**	**Category**	**Relation to the BHP role and Implementation of CCC**
**System**	**Working in the VA context**: *Specific characteristics of the VA system and Veterans that impacted the practice and professional behaviors of BHPs*	· VA's EMR and Clinical Reminders system facilitated mental health screening but could be time consuming, thereby impacting ability to provide brief treatment
		· BHPs at geographically distant and diverse CBOCs experienced logistical and travel-related barriers when referring Veterans for specialty mental health services at VAMCs
		· Patient complexity impacted BHPs ability to provide focused, brief treatment
**System and Clinic**	**Managing access to care on the front line**: *BHPs perceived role in increasing Veterans’ access to behavioral health care by providing brief assessment and intervention in primary care*	· Attending to Veterans in crisis impacted BHPs ability to maintain an open access schedule to provide population level care
		· BHPs exerted considerable effort in developing local workarounds to address wait times for specialty mental health services
	**Assessing a care trajectory***: In lieu of established clinical practice guidelines, BHPs engaged in a process of predicting the appropriate course of behavioral health care a Veteran would receive during, or in conjunction with, CCC*	· BHPs typically immediately referred to specialty mental health care those Veterans with clear patient safety risks, significant functional limitations, or patients with stated strong preferences for specialty mental health care
		· Among patients without clear indicators of need for specialty care referral, BHPs relied on clinical judgment and indicators that patient-specific goals were achieved to suggest the end of treatment
	**Developing a local integrated model**: *The combination of local resource limitations and BHP’s knowledge and skills regarding population-based care models directed how CCC was enacted at each clinic*	· The *Hybrid Clinic* combined elements of a traditional specialty mental health and minimally implemented population based model due to both limited local resources and limited BHP knowledge and skills regarding CCC
		· The *Brief Treatment Clinic* provided brief treatment limited to common mental health issues and was a product of moderate local resources and low to moderate BHP knowledge and skills regarding CCC
		· The aspirational *Truly Integrated Primary Care Clinic* provided brief treatment for both mental health and health psychology issues among clinics with high local resources and high levels of BHP knowledge and skills regarding CCC
**Clinic and Provider**	**Working in collaborative teams:***BHPs felt most satisfied with their jobs when they believed that they were contributing to a high functioning collaborative team to improve outcomes for Veterans in primary care*	· Communication with primary care staff was the single most important factor in developing working collaborations, with BHPs adopting a highly flexible stance in finding ways to communicate with medical providers
		· PCPs openness and understanding of CCC facilitated collaboration, especially when willing to co-manage patients
	**Being a behavioral health generalist:***BHPs described a "generalist" role because although they treated Veterans with mental health diagnoses, they addressed a wide variety of presenting concerns with an emphasis on improving functional outcomes*	· BHP's generalist role was comprised largely of providing brief assessment, treatment, and outcome monitoring directed by a patient-centered stance
		· Initial and on-going assessment of Veterans emphasized functional domains through clinical interview and patient report over assessment of psychiatric symptoms with formal assessment tools
		· BHPs reported using a wide range of interventions, but forms of cognitive and behavioral therapies were most commonly cited
		· BHPs believed that having significant clinical experience prior to entering the CCC environment was critical in developing accurate case conceptualization and diagnostic skills

### Category 1: working in the VA context

*Working in the VA Context* referred to several features of the VA system and the Veteran population that acted as barriers or facilitators to enacting CCC. Participants noted that the VA’s electronic medical record (EMR) was a generally beneficial part of clinical practice because it facilitated access to patient information. However, not all aspects of the EMR were considered in a positive light. Clinical reminder (CR) technology is embedded within the EMR to cue BHPs to complete required annual screenings for high frequency behavioral health concerns, such as depression and alcohol use. CRs track which patients are due for screening, prompt the BHP when the screen is due, provide scripts for completing the screen and appropriate follow-up actions, and generate documentation of the screen results. BHPs considered CRs to be both a facilitator and hindrance to providing care. On one hand, they found CRs useful for prompting them to adhere to VA clinical practice guidelines. On the other hand, the amount of time required to complete the CRs could be considerable:

…Especially a brand new patient -- you get somebody who’s really first time -- and you can spend 40 minutes doing clinical reminders…If the < alcohol use screen > is positive then you do two more things (participant A12).

In a standard 30 minute encounter, completing numerous CRs meant little time remained to address the Veteran’s presenting concern. Aside from CRs, participants also expressed uncertainty about whether other requirements typical in specialty mental health settings were also required for CCC clinics, such as conducting full psychosocial assessments or suicide risk assessments.

Participants also noted the challenge of providing a uniform set of behavioral health services across diverse clinic settings, especially CBOCs. As noted by one participant, being stationed at a CBOC was “sometimes a blessing and sometimes a curse” (A7). Working in the CBOC setting provided BHPs a greater sense of freedom because there was frequently less direct supervisory oversight, yet this freedom was commonly counterbalanced by additional challenges. One reported downside of providing services at CBOCs included that clinics had highly variable physical layouts that imposed barriers to patient care, co-location of services, or collaboration with PCPs. One BHP noted that her office location was actually located outside of the primary care clinic itself.

Perhaps the greatest impact for BHPs working at CBOCs was the geographic distance to the nearest VAMC which acted as a barrier to referring Veterans who needed a higher level of mental health care than could be provided in CCC. BHPs were well aware that many Veterans' lacked access to or funds for transportation. A BHP working at a CBOC that was several hours away from the parent VAMC noted thatTo overcome these travel barriers, BHP’s care facilitation role expanded to include the time-consuming task of exploring workarounds to connect Veterans to local, non-VA services.

…if anybody needs special services or special care or if I have a candidate that needs, maybe, substance abuse treatment or would benefit better from, say, ongoing PTSD groups, more intensive-type treatment than therapy… I have to make referrals to < nearest VAMC >, and that poses a problem for most of our patients for travel (participant A5).

In addition to characteristics of the VA itself, Veterans were perceived by BHPs to be more complex than patients in the general community, as indicated by the number of Veterans with significant medical and psychiatric comorbidities, greater levels of psychological distress, or limited social support. Additionally, BHPs reported that some Veterans appeared to enter the CCC appointment with the expectations of traditional, unstructured "talk therapy." That expectation was at odds with the constraints of working in a CCC model. Despite these complexities, BHPs enthusiastically noted their strong sense of satisfaction in helping Veterans with diverse backgrounds to get the care they deserve: "I love the patient group. I just love working with Veterans" (participant A9). BHPs felt committed to providing patient centered care, but there were clearly challenges to tailoring services to the individual while meeting administrative mandates and addressing system and logistical barriers.

### Category 2: managing access to care on the front line

*Managing Access to Care on the Front Line* referred to BHPs' perceived role in increasing Veterans’ access to behavioral health care by providing services in primary care:One participant noted the professional satisfaction of “providing care for people who normally would not access behavioral health. With everything in primary care, it’s much easier and less stigmatized to be able to see a provider” (participant A6).

But my job, of course, is front line. Meeting with patients, helping them understand access to behavioral health care…I do a lot of supportive counseling, a lot of patient education, a lot of front line – this is your first stop for many of these patients for mental health care. So trying to educate them as to the process, normalize what’s going on… (participant A4).

However, two key factors emerged that acted as barriers to BHPs ability to act as a convenient point of access to behavioral health for Veterans. First, adhering to a 30-minute hour was easily disrupted by attending to a Veteran in crisis, whether in person or by phone. One participant noted the following:

I’ve been sort of the point person for a lot of crisis cases, so I think that certainly affects my work. I think, in theory, crisis is not something that would be handled in primary care, but it has become a significant portion of my work (participant B1).

Particularly in CBOCs where the BHP was the only available mental health provider, managing patients with significant suicidal ideation resulted in considerable delays and backups in providing care to other Veterans. Thus, not only was suicide risk clearly an issue of clinical attention, it became a systems issue when alternative approaches to managing crisis, such as escorting patients to the VA emergency room or specialty walk-in clinics, were not available. Secondly, BHPs encountered barriers in maintaining access to services for patients when specialty mental health clinics had limited availability to absorb referrals from primary care. In reference to the interconnection between her CCC practice and the larger behavioral health system within the VA, another participant noted that

"… if there were more support further in the system, it would also allow us to function in the ideal model (participant A1)."

BHPs primarily developed workarounds for these system delays, including finding community referrals or providing specialty care interventions in primary care. Some BHPs reported that they referred the veteran for specialty care but continued to provide support and monitoring in primary care until the Veteran was transitioned to specialty mental health. Offering this style of interim care assured providers that the behavioral health needs of their patients were being met to an appropriate standard even though they were deviating from a population–based model if the care transition was especially protracted. BHPs tended not to engage in broader system-focused changes related to the operations of specialty mental health.

### Category 3: developing a local integrated model

*Developing a Local Integrated Model* depicted how resource limitations and BHP’s level of knowledge and skills influenced CCC at each clinic. First, resource limitations, including the physical layout of the clinic, level of BHP staffing, availability of a prescribing BHP (in person or by telepsychiatry), availability of access to specialty mental health services, and the PCPs' degree of receptivity to the BHP varied significantly from clinic to clinic. Second, BHP’s knowledge of the features of the CCC model and expertise in providing brief interventions impacted whether or not their clinics resembled an ideal form of CCC. Taken together, these factors resulted in two predominant models of partially implemented CCC as well as third aspirational model of CCC.

*The Hybrid Clinic* model reflected a marginal level of CCC implementation due to significant resource limitations and low to moderate BHP knowledge about enacting CCC. Hybrid specialty clinics were typically within CBOCs where there was very limited BHP staff within a given geographic area. In this model, BHPs attempted to provide brief treatment, but perceived the majority of the Veterans they served as too complex, and correspondingly provided longer appointments or more frequent appointments to accommodate this perceived need:

But there are just so many mental health patients out here… a lot of combat, Vietnam, long-term PTSD type of people who haven’t gotten counseling who are sort of coming out of the woodwork now. And they just are really taking up a lot of time, and you’re not going to be able to see them for a half hour, 5 sessions. It just isn’t going to work (participant A9).

Typically, only Veterans with the most severe levels of impairment were referred to specialty services with significant effort exerted on behalf of the Veteran and BHP to overcome geographic barriers to access those services.

In contrast, *The Brief Treatment Clinic* typically occurred in locations with moderate levels of clinic resources, but low to moderate BHP knowledge regarding CCC. This model emphasized providing services for most Veterans using a limited number of sessions. Triage and referral were also key components of this model because BHPs emphasized that specialty mental health care was not available in their clinic. As noted by one provider, her goal was to:

…help patients understand that this is outpatient – it is not specialty care…. My goal is to work myself out of a job. My goal is, let’s identify the issue you want to work on, let’s work toward that as best we can, and let’s get you stabilized and out of here. (participant A4).

Brief treatment targeted common mental health diagnoses rather than health psychology or behavioral medicine concerns. Additionally, a key barrier to maintaining the brief model focus was encountered when BHPs who were social workers were asked to provide medical social work services (e.g., assistance with disability claims, obtaining health insurance, addressing housing and transportation issues) in addition to mental health services.

The far end of implementation of high-fidelity CCC was reflected in BHPs’ notions of *The Truly Integrated Primary Care Clinic.* According to participants, this model of care was rarely fully implemented, but embraced the ideology that CCC should be directed at improving general health and promote both mental and physical wellness. Whereas interest in conducting this style of clinic was high, most participants noted that it was not feasible due to their limited training in behavioral medicine, the lack of referrals by PCPs for these types of concerns, and a lack of time to address these issues above and beyond the more pressing mental health concerns.

### Category 4: assessing a care trajectory

A*ssessing a Care Trajectory* was the process of predicting the appropriate course of behavioral health care a Veteran would receive during, or in conjunction with, CCC. The population-based approach of CCC suggests that a minority of Veterans would need to be referred to specialty care, yet practice guidelines for how to evaluate Veterans in this manner did not exist. Thus, at the first encounter and through the course of treatment in CCC, the BHPs looked for indicators that a Veteran would need a more intensive form of treatment offered through specialty mental health. More specifically, BHPs’ ability to assess a care trajectory was informed by past clinical experience, their understanding of system level resources, previously identified system workarounds, and attempts to meet patient preferences. Because it was common for Veterans to present with multiple concerns, BHPs looked for clinical history or symptoms that might indicate need for referral to specialty care. BHPs reported that they used the following as indicators of need for specialty care: a) a history of psychiatric hospitalizations followed by continued engagement in high risk behaviors, b) significant or chronic suicide or homicide risk, c) active substance abuse, d) multiple psychiatric comorbidities or personality features that might limit utility of short term treatment, e) the need for complex medication management, or f) interest in specialty treatment expressed by the Veteran.

However, determining a care trajectory was often more challenging when Veterans did not evidence an obvious safety risk. Thus, BHPs were left to rely more on clinical judgment in determining if the Veteran could be treated in CCC or referred out:

I am looking at this person, and I’m saying to myself, “Is this somebody who would be better served by the behavioral health clinic; are they going need years, or even months, of treatment?” Or, is this somebody, in my best guess, who is going to pop out of this with a little bit of work (participant A1).

Additionally, BHPs noted that care ideally ended when the Veteran had resumed a higher level of functioning or improved coping, even if a “cure” of the larger constellation of the Veteran’s concerns was not achieved. With CCC’s emphasis on time-limited treatment, it appeared that being skilled at evaluating and re-evaluating when to discontinue care was crucial for managing one’s caseload.

### Category 5: working in collaborative teams

*Working in Collaborative Teams* referred to BHPs' expressed desire to contribute to high functioning interdisciplinary primary care teams. Successful clinics were described as having a good rapport and sense of community among providers. Communication with primary care staff emerged as the single most important factor in developing collaboration. BHPs reported adopting ways to communicate that were in line with PCP preference, but desired in-person communication that facilitated care and created more opportunities to provide immediate services to Veterans. Additionally, BHPs facilitated referrals from PCPs by being open to interruptions, or having an open door policy:

"I’m here to assist their primary care provider in giving them the best care in a holistic way. And I talk to them about a real focus on access that we try to provide. Much of what I do is same-day consultation, where the primary care provider walks the patient over to my office or I will sometimes sit in there with them with patients. So I don’t sit and wait for the business to come to me, so to speak. I really try to be consistently reminding them of my role, to be available" (participant A7).

BHPs also noted that those PCPs who showed enthusiasm for CCC clearly facilitated collaboration. PCP acceptance of CCC indicated that they were showing willingness to co-manage patient needs. One BHP described her past experiences with PCPs who were unwilling to refer Veterans to her or consider prescribing psychotropic medication:

"I’ve been more dissatisfied with my role when primary care has been more resistant to embracing that model of care. They either don’t refer anybody, or they want you to take care of everything, and not co-manage people together" (participant A3).

BHPs offered several reasons for this, but primarily noted that some PCPs were either new to the VA system or did not understand the BHP role in CCC. Additionally, some PCPs appeared to be experiencing role strain and did not understand the assistance a BHP could offer.

### Category 6: being a behavioral health generalist

BHPs described their role as that of a behavioral health generalist who provides assessment, treatment, and outcome monitoring, ideally directed by a patient-centered stance. Depression and posttraumatic stress disorder were the most common reasons for referral, but as one participant suggested, "…the behavioral health provider is expected to take care of basically the full range of problems” (participant C1). During initial appointments functional assessment was a primary goal for the BHP, although most participants reporting using standardized instruments to assess psychological distress infrequently.

Assessment could be a lengthy process, but highly experienced BHPs relied on their past experience to efficiently formulate clinical impressions. Skilled BHPs also rapidly gleaned key diagnostic, functional, or medical information from prior behavioral health screenings or progress notes. The specific interventions BHPs stated they used were diverse and selected based on the information identified in the initial functional assessment. Interventions derived from empirically supported methods such as cognitive-behavioral therapies were most commonly reported. BHPs also noted the importance of providing a "take-away,” or a positive behavioral health experience that improved Veterans' knowledge or coping skills that could be applied outside of the CCC encounter.

Monitoring Veterans' response to treatment was focused on changes in function relative to the Veteran's stated goals. Data for assessing outcomes consisted primarily of behavioral observations and Veteran self-report. Outcomes commonly reflected changes in adopting healthy behaviors, stabilization of distress symptoms, a decrease in unnecessary primary medical care utilization, or improved general functioning. A participant gave the following example of common patient-centered behavioral goals:

"We look at functional outcomes, you know they’re walking more, they’re going to work, they’re not missing work, they’re doing social activities, as I said some of those medical parameters are under better control" (participant A7).

Participants who were most satisfied in their position were those with the most clinical background prior to entering their role in CCC. BHPs mentioned the advantage of having worked in medical environments, prisons, substance abuse specialty programs, serious mental illness treatment settings, as well as outpatient specialty mental health clinics. One participant specifically cited the value of social work training:

"I think because it is a holistic approach. And I think that coordinating services in an integrated system includes a need to be holistic" (participant D1).

Skilled generalists were able to draw from well developed clinical sensitivities, apply a toolbox of interventions, and work rapidly. Finally, health psychology background was considered quite useful in CCC, but BHPs lack of experience in this area was often mentioned as a clear barrier to providing such services.

## Discussion

This secondary analysis of qualitative interviews collected as part of an integrated care quality improvement project identified system, clinic, and provider level barriers and facilitators to implementing CCC. We emphasized exploring insiders' perspectives on usual practice patterns in the hopes of describing the on-the-ground reality faced by BHPs in primary care. At the time of data collection, most BHPs had assumed their role in CCC with implementation support limited to brief consultations with integrated care experts. On-going implementation interventions, such as the use of facilitation, have been suggested to improve adoption of clinical innovations [23-25] were not routinely employed at the time. BHPs reported that their behaviors related to CCC were based largely on pre-existing knowledge or assumptions regarding how CCC should be practiced, while also trying to balance administrative requirements that were not always consistent with the CCC model. BHPs typically learned on the job by making necessary changes to practices developed as specialty mental health providers.

Despite limited support, BHPs in this study reported several encouraging practices that enabled CCC implementation. Foremost, BHPs reported that they valued keeping a patient-centered focus, felt a dedication to working with the Veteran population, and often extended considerable effort to ensure Veterans were connected to services that best suited their needs other than CCC. BHPs showed an awareness of system level factors and their role in the larger VA behavioral health system, including a desire to be team players and work on par with other primary care staff. They also engaged in self-directed learning by reviewing professional journals, attending conferences and trainings, and increasing their understanding of empirically supported treatments typically used in specialty mental health settings, even though these modalities required significant modification for use in the CCC environment.

Because VISN 2 was an early adopter of CCC, results from the current study are especially timely as VA is actively engaged in a process of fully implementing PC-MHI across the country [26]. This study therefore provides an opportunity to examine both how CCC evolves with limited systematic implementation support, as well as a chance to direct future interventions that may improve implementation of CCC. Furthermore, study results are no less relevant for clinics that have achieved some degree of CCC implementation but may benefit from quality improvement initiatives to enhance model fidelity and sustainability.

### Implications for training, implementation, and future research

BHPs in CCC are increasingly the first point of contact for Veterans in need of mental health services [18]. As such, it is vitally important that they are well prepared and supported to address the mental and behavioral health needs of this population. As noted within the category of *Being a Behavioral Health Generalist,* BHPs commonly treated a wide variety of presenting concerns with an emphasis on improving functional outcomes. There was also repeated mention of the increased need of these BHPs to be equipped to address behavioral medicine, or health psychology, in addition to the mental health needs of Veterans. Given the many demands on BHPs, these providers could benefit from additional tools to support their broad-based clinical practice. Some clinical support tools can be provided with relatively few resources, such as supplying BHPs with high quality patient education handouts on frequently discussed topics. Although a seemingly minor issue, busy providers may find it challenging to set aside time during busy clinic days to develop such materials. Likewise, BHPs in CBOCs often referred Veterans to community-based resources to circumvent travel barriers or to provide an alternative to VA care if specialty care was otherwise difficult for Veterans to access. Given that many Veterans already rely on both VA and non-VA primary and specialty medical care [27,28], developing local, community-specific lists of non-VA mental health and substance abuse services may be helpful for both providers and patients.

Perhaps the largest area in which BHPs could use additional support relates to intervention and assessment options. BHPs frequently expressed the pressure to balance shorter appointment length and fewer visits with the provision of evidence-based interventions and measurement-based care. Studies have repeatedly demonstrated that patients visit a PC-MHI provider, on average, 2–3 times [14,16,18]. Currently, the only robust evidence base for treatment with such limited number of appointments is for the brief treatment of Alcohol Use Disorders [29]. In contrast, the briefest well studied treatments for depression, the most common condition reported in primary care, require 6 to 8 sessions [30]. Future research needs to address this paucity of evidence for very brief interventions that are practicable for CCC. In the interim, BHPs may need additional support in adapting specialty care based, full-length, empirically supported treatments for use in the primary care setting.

Within the category of *Assessing a Care Trajectory*, it was suggested that without established guidelines, BHPs relied on clinical judgments, identification of high risk features, and subjective indicators of goal attainment to guide termination or referral to a higher level of care. This category uncovered a tacit link that existed between BHP’s skills in individualized assessment and their system level understanding of how to proceed with patient care. The BHP aimed to strike a balance between referring to a higher level of care and adhering to a population-based approach in which ideally only a minority of patients are referred to specialty mental health. Assessing a patient’s trajectory was essentially an on-going process which, while remaining patient-centered, helped maintain a brief treatment model by rapidly identifying and referring out those who would be in need of a higher level of care. Although BHPs intuitively engaged in this process with some success, CCC programs would likely benefit from developing referral algorithms, clarifying which Veterans should be seen in primary care, and which, if agreeable should be referred to specialty mental health clinics. Furthermore, clearly articulated service agreements across CCC, primary care, and specialty mental health clinics could address a range of clinical issues that are especially challenging to BHPs, such as standardizing procedures for managing crisis interventions and transitioning patients to *and from* more intensive specialty mental health services. While specific details would need to be adjusted based on site resources and distance to the specialty mental health services, general clinical guidance on limitations expected in PC-MHI programs would be helpful to managers trying to successfully direct resources and fully implement programs. New BHPs with minimal clinical experience in CCC may find such guidance especially helpful in maintaining a population-based approach.

As noted in the *Developing a Local Integrated Model* category, local resource availability also contributed to differences in how CCC was conducted at each site. The predominant model of CCC identified, *The Brief Treatment Clinic,* illustrated the tension of BHPs exerting effort to develop an integrated clinic model without clear systemic support that might ensure higher fidelity to CCC. Systems with rich resources might facilitate a BHP’s adherence to components of a population-based model ultimately leading to *The Truly Integrated Primary Care Clinic*. On the other hand, providers embedded in over-taxed behavioral health systems are likely to struggle to reach high levels of CCC fidelity.

Attention to team development may also aid BHPs as noted in the *Working in Collaborative Teams* category. BHPs valued open lines of communication with primary care teams and described considerable flexibility in finding ways to communicate with PCPs regarding basic elements of patient care. Although there are many factors that influence team dynamics and cohesion, BHPs can strengthen their collaborations with primary care teams by consistently demonstrating their value as clinicians. BHPs can provide education to the team regarding their unique role in primary care as behavioral health generalists who provide a wide range of services, including linkage to other VA and community resources. PCPs may logically infer that BHPs are suited for primarily addressing mental health concerns among Veterans. As noted previously, improved skills related to behavioral medicine and health psychology might provide an opportunity for BHPs to engage more fully with primary care teams on common presenting concerns among Veterans in primary care, such as hypertension, diabetes, obesity, or chronic pain. Taken together, BHPs who can provide patient-centered intervention that addresses mental health concerns as well as the behavioral and psychological aspects of chronic disease may ultimately engender trust and buy-in from PCPs as demonstrated by greater rates of consultation and co-management of patients.

### Study limitations

This study has several strengths and limitations to consider when interpreting the findings. These data were collected prior to the implementation of care management in VISN 2, so this analysis fails to provide information about the experience of CCC staff in relation to care management. As VA requires both CCC and care management at all VAMCs and large CBOCs, PC-MHI staff experiences related to blending CCC and care management will be important to address in future studies. In addition to PC-MHI, VA is currently engaged in a large scale reorganization of its primary care services referred to as Patient Aligned Care Teams (PACT), modeled after the medical home movement that has gained such positive recognition outside VA [31]. Data collection for the current study occurred well before the initiation of PACT in VISN 2 and thus cannot address the added complexity or benefit of its implementation activities on PC-MHI programs.

Additionally, it is important to consider that although conducting a rigorous qualitative analysis maximized the usefulness of previously collected data, we were limited in our ability to engage in theoretical sampling often employed in grounded theory that would have better shaped the resulting categories. Typically, data collection in a grounded theory study is complete when saturation is achieved, or when additional data analysis yields no new concepts, but deepens or refines previously developed categories. In the case of this secondary data analysis, although there was sufficient data to reach saturation [32] with well-developed categories, we were limited in our ability to generate a mid-range theory through theoretical coding.

## Conclusions

BHPs in CCC settings play an essential function in the VA health care system by improving access to mental health services for Veterans in primary care. The study offered an experience-near perspective from BHPs that identified both facilitators and barriers to implementing CCC at a time when minimal formal implementation support was available. Although BHPs described their role as generalists, analysis revealed a set of duties and practices that were decidedly complex, often demanding, and wide-ranging in scope. Future implementation efforts should address an equally wide range of targets, such as enhancing providers’ clinical skills in relation to delivering population-based behavioral health interventions. Additionally, implementation support can focus on fostering strong professional relationships between BHPs and members of primary care teams that will pave the way for increased collaboration. In the same way that BHPs aimed to provide patient-centered clinical care, it will be paramount to offer provider-centered implementation supports to enhance BHPs enactment of CCC.

## Abbreviations

BHP: Behavioral Health Provider; CR: Clinical Reminders; CCC: Co-located, Collaborative Care; CBOC: Community Based Outpatient Clinic; EMR: Electronic Medical Record; PACT: Patient Aligned Care Team; PC-MHI: Primary Care-Mental Health Integration; PCP: Primary Care Provider; VA: Veterans Administration; VAMC: Veterans Affairs Medical Center; VISN: Veterans Integrated Service Network.

## Competing interests

There are no known conflicts of interest for reasons financial or otherwise, no known competing interests, and no companies or products are being featured in this research.

## Authors' contributions

GB conceived of the study and LW assisted in developing its design. GB conducted the primary data analysis, with subsequent input from LW regarding finalization of categories. GB and LW drafted the manuscript, and both authors read and approved the final manuscript.

## Pre-publication history

The pre-publication history for this paper can be accessed here:

http://www.biomedcentral.com/1472-6963/12/337/prepub
